# Prosodic Structure as a Parallel to Musical Structure

**DOI:** 10.3389/fpsyg.2015.01962

**Published:** 2015-12-22

**Authors:** Christopher C. Heffner, L. Robert Slevc

**Affiliations:** ^1^Program in Neuroscience and Cognitive Science, University of Maryland, College ParkMD, USA; ^2^Department of Linguistics, University of Maryland, College ParkMD, USA; ^3^Department of Hearing and Speech Sciences, University of Maryland, College ParkMD, USA; ^4^Department of Psychology, University of Maryland, College ParkMD, USA

**Keywords:** musical structure, prosody, prosodic structure, music perception, speech perception

## Abstract

What structural properties do language and music share? Although early speculation identified a wide variety of possibilities, the literature has largely focused on the parallels between musical structure and syntactic structure. Here, we argue that parallels between musical structure and prosodic structure deserve more attention. We review the evidence for a link between musical and prosodic structure and find it to be strong. In fact, certain elements of prosodic structure may provide a parsimonious comparison with musical structure without sacrificing empirical findings related to the parallels between language and music. We then develop several predictions related to such a hypothesis.

## Introduction

Uncovering structural similarities between language and music has been an objective that has tantalized cognitive scientists for years. Music and spoken language both consist of complex auditory signals that are organized in line with an underlying structure. But which linguistic structures are musical structures parallel to? In their seminal work proposing a generative account of musical structure, Lerdahl and Jackendoff (1983, p. 330) concluded that “the similarity between prosodic and music structure can be used as a point of triangulation for approaching an account of other temporally structured cognitive capacities.” In other words, they argued that musical structure and prosodic structure (patterns of rhythm, pitch, and tempo in speech) have convincing parallels, worthy of study and comparison to each other. Although it is certainly the case that subsequent investigations of the links between phonetic processing and musical structure have often relied on the idea of a music/prosody connection (Schön et al., 2004; Moreno et al., 2009; Besson et al., 2011), most subsequent work comparing musical structure to language has focused on comparisons with linguistic syntax (i.e., the combination of words into sentences) and not prosodic structure.

Here, we revisit Lerdahl and Jackendoff’s (1983) speculations about the similarities between the structure of music and the structure of prosody. We first review the literature connecting music and linguistic syntax, finding that evidence for certain parallels between the two modalities (especially with regard to recursion) surprisingly sparse. We suggest that this opens up opportunities to examine prosodic structure as another fitting analog to musical structure. We describe prosodic structure, highlighting the ways that it differs from syntactic structure, as well as its many similarities. We suggest that comparing musical structure to prosody does not require discarding syntactic parallels, and highlight some comparative insights that could be gained by additionally examining prosody. Taking prosodic structure seriously, we argue, also helps tie together a few disparate streams within the experimental literature that are currently unrelated. Finally, we make some predictions for future studies that would be in line with a prosodic account of musical structure.

Although the purpose here is to accentuate the parallels between musical structure and prosodic structure, we do not mean to imply that musical processing does not have multiple parallels to language processing. It is certainly the case that comparisons between music and other linguistic systems are useful. However, in this review, we do seek to point out some of the ways that it is unfortunate that the use of prosodic structure to understand musical structure has fallen by the wayside. Studies that have analogized musical structure to syntactic structure often serve as a useful counterpoint in these discussions, particularly concerning the questions that are left unresolved or incomplete by a syntactic analysis of musical structure.

## Music and Syntax

### Evidence for Syntactic Parallels

Most of the discussion surrounding structural parallels between music and language has focused on using theories of syntactic structure to bridge the two fields. This may have something to do with the prominence of syntax within the language sciences, where it is undoubtedly accorded a place of honor. Indeed, although the idea is not shared by every language scientist, the recursive structure of syntax has been argued to be the attribute that separates human language from other communication systems (Hauser et al., 2002). Syntactic recursion allows syntactic structures to be embedded inside other syntactic structures, which can, in turn, be embedded inside others. This embedding process creates a hierarchical structure where certain parts of the structure are privileged over others and sometimes reduplicated at higher levels. Syntactic recursion is a powerful property because it allows speakers to potentially utter an infinite number of sentences.

Both music theorists and language scientists who have music-related interests agree that aspects of music can be described in terms of recursive formalisms similar to syntactic structure (although we note that this discussion has centered almost entirely on Western tonal music). These syntactic structures can be used to assemble a generative grammar of music. Such generative grammars for musical sequences have been proposed for jazz chord sequences (Steedman, 1984), tonal harmony (Lerdahl, 2001b; Rohrmeier, 2011), and rhythmic structure (Longuet-Higgins, 1976), and have often reflected contemporary conceptions of linguistic syntax (Katz and Pesetsky, 2009). Such ideas may have emerged in part from a long tradition of recursive structural analyses of music and from the influence of recursion on musical composition (Forte, 1959; Larson, 1998). This historical framework may similarly have influenced the argument that syntactic processes play a central role in the cognitive processing of music (e.g., Patel, 2003, 2008; Koelsch, 2005).

Indeed, there is evidence for music/syntax relationships in the experimental literature. For example, rhythm perception can predict syntactic skills among typically developing 6-year-olds (Gordon et al., 2015; see also Jentschke and Koelsch, 2009). Other work suggests similarities between the neural correlates of musical and linguistic structural manipulations; that is, event-related potential (ERP) components associated with syntactic phrase violations have often been uncovered in electroencephalography (EEG) studies that have investigated musical phrase structure (Patel et al., 1998a; Koelsch, 2005).

This is true not just for short-distance musical dependencies, but also ones that are spread across multiple musical phrases. For example, Koelsch et al. (2013) found EEG evidence for listeners’ sensitivity to hierarchical structure by manipulating passages taken from two Bach chorales. Each chorale originally had an ABA structure, with information in the last phrase resolving musical structures left incomplete by the initial “A” phrase. In their experiment, Koelsch et al. (2013) contrasted the original chorales with modified versions with a CBA structure; that is the first “A” phrase was pitch-modified such that the final passage did not resolve the musical structures left incomplete in the initial passage. Because the pitch manipulations involved were performed on the initial phrase, not on the final chord, any ERP response differences to the final chord could not have been the result of an acoustic difference between the two stimuli. There were indeed ERP components that differentiated the two conditions, which they interpreted as evidence for sensitivity to hierarchical syntactic structure in music.

Other work localizes the neural correlates of musical structural violations to regions often implicated in syntactic structural violations, such as Broca’s area, although such parallels may depend on the task demands of the experiments in question (LaCroix et al., 2015). Nevertheless, neural correlates of musical structural violations have also been found to resemble those associated with syntactic structural violations in both magnetoencephalography (MEG; Maess et al., 2001; Koelsch and Friederici, 2003) and functional magnetic resonance imaging (fMRI) studies (Tillmann et al., 2006; Oechslin et al., 2013; Seger et al., 2013).

### Challenges for Syntactic Parallels

While this work supports a relationship between musical structure and linguistic syntax, there are both theoretical and empirical reasons to question the idea that musical structure and syntactic structure in language are fundamentally similar. First, some empirical work has failed to find overlapping regions associated with structural manipulations in music and language within individual participants (Fedorenko et al., 2011, 2012; Rogalsky et al., 2011). Fedorenko et al. (2011), for example, presented participants with sentences and lists of non-words (both presented word-by-word) and identified regions within individual participants that selectively responded more to sentences than to non-word lists. Many of these regions were in left frontal and temporal cortices (e.g., left inferior frontal gyrus, left anterior temporal cortex). They then had participants listen to music and scrambled music, and found that the regions that showed significant difference in activation between sentences and non-word lists were not consistently more activated by music more than scrambled music. Fedorenko et al. (2012) performed essentially the converse experiment. First, they identified regions within individual participants that were sensitive to music over scrambled music; for most participants, this included bilateral temporal cortex and supplementary motor area. They then had participants view sentences, word lists and non-word lists. They found that the regions that were more activated by music than scrambled music did not show differential activation to each individual kind of sentence stimulus.

More generally, many fMRI studies of syntax/music connections suffer from challenges in differentiating shared patterns of fMRI activation and shared underlying circuitry (see Peretz et al., 2015, for discussion). Furthermore, the evidence for interactive effects between musical and syntactic manipulations (Carrus et al., 2013; Kunert and Slevc, 2015) is complicated by evidence for interactions between musical structure and non-syntactic linguistic variables (Perruchet and Poulin-Charronnat, 2013) and even between musical structure and non-linguistic processes (e.g., Escoffier and Tillmann, 2008).

Finally, and perhaps more importantly, we argue that the evidence in favor of recursion being used in musical processing is scant. On the face of it, this may seem like a surprising claim. Composers, after all, do often include higher-level structure in their pieces; consider the rondo in many concerti (with an ABACABA structure) or variations on verse-and-chorus structure in popular music. Yet the fact that these structures exist does not necessarily mean that listeners are sensitive to them in day-to-day listening situations. Studies that have carefully investigated the perception of this long-distance structure have indicated that listeners, even ones with musical training, may simply ignore long-distance structural dependencies (Cook, 1987). For example, when listeners were asked complete a “musical puzzle”—that is, to organize parts of a minuet into a single, coherent piece—listeners often violated the typical structural relationships between each component part when constructing a piece (Tillmann et al., 1998). Although findings have shown evidence for some implicit learning of structural properties of language within a short time-window, the evidence for long-distance processing of dependencies is much weaker (Tillmann and Bigand, 2004). Studies that have assessed such processing have generally not used implicit measures that might indicate implicit learning of those sequences, however. Such findings suggest that listeners do not routinely extract recursive structure from a musical signal without explicit, post facto scrutiny.

These findings that listeners are not typically aware of long-distance musical structure conflict with other evidence taken to show recursive processing of musical structure. For example, Koelsch et al. (2013) found that participants’ EEG responses differentiated regular from irregular resolutions of long-distance dependences in music (as described above). However, it may be that these data do not actually reflect recursive processing of musical structure, but instead reflect listeners’ expectations about the timing and pitch information found within a piece rather than something fundamental about musical structure. This is plausible because, in Koelsch et al.’s (2013) experiment, participants listened to regular and irregular versions of only two different Bach chorales (transposed into each major key) 60 times each, presented in a randomized order. By the end of the experiment, it was likely that the rhythmic and pitch properties of these pieces became very well learned. Participants could therefore use information (explicitly or implicitly) early on within the chorale to predict future musical patterns. Alternatively, the listeners might simply be using the time they had been spending listening to the renditions of each version of each chorale to think about the music in a more abstract (and potentially recursive) way. Thus, these results are not inconsistent with the idea that listeners do not *normally* engage in recursive processing of musical structure during typical listening.

## Prosodic Parallels

### Structural Description

**FIGURE 1:**
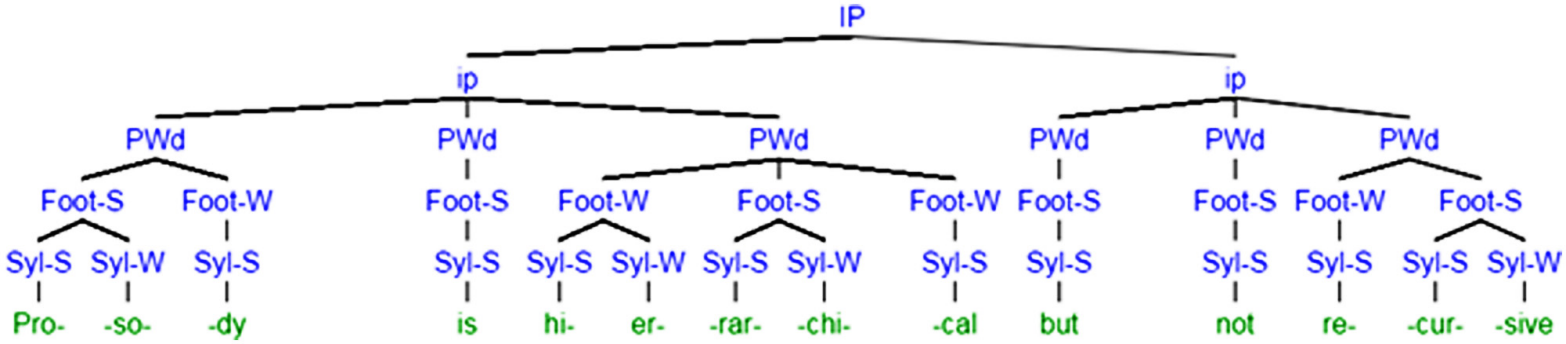
**Prosodic description of the phrase “Prosody is hierarchical but not recursive.”** Prosodic tiers are organized across rows. For the syllable and foot tiers, some syllables or feet are labeled strong, while others are labeled weak. This allows for differences in stress across words. Tree generated using Miles Shang’s Syntax Tree Generator, available at http://mshang.ca/syntree/.

Given these challenges for syntactic parallels to musical structure, we argue here that prosodic structure may better parallel the structure of music. The first thing to observe about prosodic structure is that it, like syntactic structure, is hierarchical. Indeed, a reader merely glancing at the prosodic tree in **Figure 1** should be forgiven for thinking that it is a syntactic tree. However, rather than phrases being different parts of speech, each unit is instead a level on the prosodic hierarchy. From bottom up, syllables are combined to form feet, which are combined to form prosodic words, which in turn are combined to form minor and major intonational phrases. This particular tree is not meant to be reflective of any single school of thought related to the organization of prosodic structure, of which there are many. A discussion of these differences is outside the scope of this paper, but almost all agree on the details of prosodic structure we bring up here. Rather, we intend the description below to be an approximate sketch of commonalities between schools of thought within prosody. Interested readers should refer to Shattuck-Hufnagel and Turk (1996), which remains the best explanation of the prosodic hierarchy to novices.

From the very base of the hierarchy (phonemes, or speech sounds), smaller units are organized into larger groupings. These groupings in turn form larger ones. And the smallest groupings can be said to “belong” to higher level groupings by way of those intermediate levels. Some of these levels might be familiar, such as syllables and prosodic words (which usually, though imperfectly, correspond to an intuitive notion of “word”). Others might be unfamiliar outside of the realm of poetry, like the “foot”, which syllables combine together to create. But crucially, each of these structures plays a role in organizing the timing, pitch, and volume characteristics of the speech signal. In many languages, for example, syllables gain stress through an increase in some combination of volume, pitch, and length on the stressed syllable (Gay, 1978). The ends of intonational phrases and prosodic words are characterized by the lengthening of phrase-final material (Wightman et al., 1992; Byrd et al., 2006). Even newborn infants seem capable of discriminating short utterances with prosodic boundaries from short utterances without them (Christophe et al., 2001).

Similarly, within music, notes can associate to form beats, measures, and phrases, each of which has consequences for the timing, pitch, and volume characteristics of the notes in question. The structure and expression of musical phrases is sometimes referred to as “musical prosody” (see, e.g., Palmer and Hutchins, 2006) and shows many parallels with linguistic prosody; for example, phrase-final lengthening is found in music as well as speech (Large and Palmer, 2002). These predictable regularities in pitch, loudness, and duration information are tracked in real-time, and have been associated with an ERP component known as the “closure positive shift” (Steinhauer et al., 1999; Steinhauer and Friederici, 2001). This prosodic boundary-making can even sometimes lead listeners to misparse the syntactic content of sentences, as revealed, for example, through ERP components that typically manifest in response to syntactic anomalies (Steinhauer et al., 1999), causing some to claim a central role for prosody in sentence comprehension (Frazier et al., 2006).

The example in **Figure 1** shows that, besides being in the onset of the syllable “hi,” the phoneme /h/ within the word “hierarchical” also belongs to the word-initial foot “hier,” the prosodic word “hierarchical,” the minor intonational phrase “Prosody is hierarchical,” and to the major intonational phrase “Prosody is hierarchical but not recursive.” There are many prosodic structures that a syllable can map onto, but the mapping is essentially exhaustive. Like in prosody, syntactic structure is often posited in tree-like terms, with units contained in other units. Like syntax, too, it is generally non-overlapping. The sound /h/ does not also, say, belong to the end of the previous syllable, /Iz/. Nor do phrases within each level of the hierarchy cross, with /h/ belonging to the “hi” syllable but not to the “is” foot.^1^ Again, this echoes syntactic structure, which has similar constraints against units that straddle higher-level boundaries.

The primary difference in description of prosodic and syntactic structure is in terms of the non-recursive nature of prosodic constituents. Recursion in syntax is, without a doubt, important; to some, it is absolutely essential (Hauser et al., 2002). Within syntax, sentences can be nested inside of sentences, adjectives inside other adjective phrases. This allows for the great complexity of human speech; for example, an infinite number of utterances can be created and comprehended by listeners who are perfectly capable of understanding sentences that have embedding. Recursion requires comprehenders to keep track of a wide variety of current dependencies and possible future dependencies based on the signal that they have heard so far, and these dependencies might be of exactly identical style. Prosody, in contrast, has no such recursive elements to it. There is no sense in which a prosodic word can be embedded within another prosodic word, or where a syllable is separated from another syllable by a few levels within hierarchy; one level of the structure is not duplicated anywhere else. This does not necessarily make prosodic structure any less rich. It simply makes it less self-embedded.

### Prosodic Correspondences with Musical Structure

Prosodic structure shares some useful commonalities with musical structure. Like prosody, it includes phrases across a variety of time scales, with shorter-duration units nested inside larger ones. These phrases have more or less prominent units, and within phrases there are often restrictions on which segments can co-occur. And, in both cases, the structures that are imposed on the signal are capable of certain prescribed exceptions.

Take meter as an example (Lerdahl, 2001a; Jackendoff, 2009). Within musical theory, meter refers to regularly recurring sequences of more or less prominent beats across time. Within prosodic theory, meter refers to more or less prominent (i.e., stressed or unstressed) syllables across time, usually in terms of the organization of syllables into feet and feet into words. In both modalities, there is a typical rhythm that manifests itself across time, either within a piece (for music) or within a language (for prosody). For example, in common time (or 4/4 time) in music, it is often the first and third beats within a single measure that are the strongest, with the first beat receiving the most intense accent. However, a composer or musician has the option of subverting the typical metrical properties of a piece by placing accents on individual notes besides the first and third beats, or by using syncopation. This can sometimes be exploited to better sync the metrical properties of music with the metrical properties of language, which in turn leads to certain favorable behavioral and electrophysiological outcomes with simple language tasks (Gordon et al., 2011). Similarly, typical metrical prominences and organizations vary in their manifestation across the world’s musical traditions. Balinese gamelan, for example, keeps track of the metrical beat through the use of an instrument called the *kajar*, which unambiguously (or, at least, unambiguously to the other gamelan players) sets out the location of each beat (McGraw, 2008).

In prosody, meanwhile, languages differ in how stress is assigned, but almost all show regular patterns; for example, they might assign stress to every other syllable starting from the last syllable within a word. Many languages allow for exceptions in the typical assignment of stress to syllables. Although all languages share some underlying structural properties, languages also have quite a bit of leeway in the realizations of that structure. In Czech and Polish, stress assignment is without exception (word-initial for Czech, penultimate for Polish), just as the *kajar* unambiguously determines the beat in gamelan music; in German, stress depends on the “weight” of the syllable in question, but tends to fall within the last three syllables; in Russian, it can occur anywhere at all. Thus, the two systems evince very similar properties to each other, including flexibility in broader organizational principles across traditions.

There is evidence that musical composers, and singers, are implicitly aware of these organizational parallels. The study of textsetting (the process of assigning syllables to individual notes in sung music) provides a useful guide. Textsetting has been described using a generative model that attempted “to achieve the best possible fit between the abstract structures representing rhythmic and linguistic surfaces” (Halle and Lerdahl, 1993, p. 7). Musical compositions seem to respect the structures of prosody composition in determining which notes are assigned which durations or which levels of accent (Palmer and Kelly, 1992). And singers spontaneously asked to chant simple sentences seem to assign syllables to beats in a way that respects their prosodic structural properties (Hayes and Kaun, 1996).

Another analogy can be drawn between musical modes or key systems and certain prosodically constrained phonological patterns, including vowel harmony. Musical modes constrain the set of pitches that are considered musically valid within a single piece or even a single phrase; valid notes within the key signature of G major are G, A, B, C, D, E, and F-sharp. Similarly, under phonological rules such as vowel harmony, the set of possible vowels that could be considered linguistically valid within a certain word is reduced. New suffixes that are attached to a word must maintain certain qualities in line with previous vowels in the word. For Turkish, a prodigious user of vowel harmony, certain vowels must share lip rounding and tongue position features within a single word (for example, the second vowel of *gördün*, “you saw,” is *ü* rather than *u*, *i*, or *ı* due to vowel harmony). Thus, in both modalities, the licit presence of certain subunits (vowels for language, pitches for music) inside longer sequences (words for language, musical phrases for music) is conditioned by constraints on co-occurrence within a larger sequence.

Even languages that do not display evidence for vowel harmony show examples of these constraints on co-occurrence within the prosodic domain of the word. In English, for example, the plural suffix has three phonetic forms: [s] as in “cats”, [z] as in “dogs”, and [ǝz] as in “horses”. Similarly, the past tense in English has three parallel phonetic forms: [t] as in “liked”, [d] as in “loved”, and [ǝd] as in “wanted”. Which variant appears in each form depends crucially on a constraint against consonants with dissimilar voicing appearing adjacently within words, as well as a constraint against consonants with a similar place of articulation appearing adjacently. These strict limits on adjacent co-occurrence resemble strict constraints on adjacent co-occurrence for the notes in Indian *ragas*, where there are often limits on, for example, which notes are “valid” parts of ascending or descending melodies within a piece of music (Castellano et al., 1984).

Nevertheless, there are exceptions that are tolerated in both modalities. Western music uses accidentals (sharps, flats, and naturals) in order to allow for a “banned” note, while almost every language with vowel harmony also has words that include exceptions to the general pattern of the language (the last vowel of the Turkish word *görüyor*, “you are seeing,” does not harmonize with the previous two vowels in the word because the present progressive suffix invariably uses the vowel *o*). And both music and language show variation in these properties. Languages that have vowel harmony may differ in which vowels undergo harmony, as well as what features of the vowel harmonize with previous ones. Likewise, musical structure varies from culture to culture, with patterns of greater typological frequency but not necessarily strict universals (see, e.g., Savage et al., 2015). All considered, it appears that prosody and music are well-aligned and, remarkably, even exhibit a similar flexibility within and across cultures.

### Prosodic Structure and Syntactic Structure

Many previous findings arguing for a relationship between music and linguistic syntax can also be used to justify the idea of relationships between music and prosody. This is true because syntactic and prosodic structure are, in many respects, closely related. For example, patterns of pitch and syllable length (i.e., prosodic structure) aid participants in uncovering syntactic structure in an artificial language learning task (Langus et al., 2012). Indeed, prosodic boundaries are typically found near syntactic boundaries. Some theories even suggest that prosodic boundary placement is *dependent* on syntactic phrases, with prosodic phrase boundaries only being possible at the beginning or end of a syntactic phrase (Selkirk, 1984). Given the reciprocal nature of these two linguistic dimensions, it might be that many structural manipulations in studies of musical structure actually reflect changes to prosodic structure, not necessarily to syntactic structure.

The roles of syntactic and prosodic structure are likely indistinguishable in many previous studies, perhaps because of how closely the two types of structure are tied. Many studies that have revealed interactions of musical structure with syntactic manipulations have also included manipulations of prosodic structure by dint of their auditory presentation (Fedorenko et al., 2009; Sammler et al., 2013). That is, because spoken sentences include patterns of pitch, rate, and volume that characterize prosody, manipulating the sentences in a way that affects syntax (by, say, scrambling them) generally also involves a concurrent manipulation of prosody (as re-ordering words implies also re-ordering the locations of pitch excursions, rate changes, and volume modulations). Similarly, studies of production that have found overlapping activation for music and language would by their nature have required the listener to create and deploy prosodic structure to produce sentences (Brown et al., 2006). Therefore, the erstwhile syntactic manipulations in such studies may actually just reflect the prosodic structures that are being built during the course of sentence production. Even self-paced reading studies (Slevc et al., 2009), which on the surface appear to divorce syntax from prosody, may not necessarily do so. Indeed, self-paced reading times correlate with prosodic structure (Koriat et al., 2002; Ashby, 2006) and self-paced reading elicits some of the very same prosody-related ERP components that show up in auditory speech perception (Steinhauer and Friederici, 2001).

How might these two similar structures be dissociated from one another? One possibility is to divorce syntactic cues from prosodic cues. Although some studies that have done this successfully in EEG have still exhibited significant effects of syntactic structure (discussed elsewhere in this paper), in many cases when using prosody-free presentation of language (e.g., isochronous visual word-by-word presentation), manipulations of syntactic and musical structure show non-overlapping patterns of activation in fMRI (Fedorenko et al., 2011, 2012). Similarly, studies that have crossed non-prosodic linguistic manipulations of semantic expectancy with musical harmonic violations in sung music have failed to find an interaction between each type of violation, suggesting that the linguistic semantic and musical harmonic systems are independent of each other (Besson et al., 1998; Bonnel et al., 2001 but see Poulin-Charronnat et al., 2005).

Even manipulations of long-distance musical information may fit into a prosodic framework. Prosodic structure also allows for the incorporation of long-distance information despite its non-recursive nature. Long-distance prosodic information has been shown to be informative to word segmentation, both in terms of long-distance pitch patterns (Dilley and McAuley, 2008) and long-distance rate information (Dilley and Pitt, 2010). Similarly, long-distance pitch and timing information can play a role in the processing of musical timing judgments (Povel and Essens, 1985; Boltz, 1993; McAuley and Kidd, 1998). However, critically, this long-distance information is not necessarily *recursive*. Incidental music in movie soundtracks, for example, quite often lacks any sort of long-distance structure or melody, but its ability to accentuate the action or mood on the screen makes it indispensable in modern movies (Boltz, 2004), while also being music that can be successfully marketed on its own. Listeners’ sensitivity to long-distance information in music may instead reflect the gradient processing of relative timing or pitch properties or the manipulation of expectations about rate or pitch information.

### Emotional Aspects of Language and Music

Musical links to prosodic structure also allow for a variety of phenomena that are presently treated as unrelated curiosities to all be motivated by the same root causes. One notable example is the connection between the emotional content of speech and music. It is probable that most researchers reading this paper have largely seen prosody discussed in the context of emotion. Prosody is essential for the comprehension of emotion in speech, which fits nicely with the broader links between musical and prosodic structure we advocate here. There is no question that speech is capable of conveying rich emotional information, above and beyond the lexical content of the words in question, and that much of this information is conveyed through patterns of pitch, duration, and volume discussed in the context of prosody (Williams and Stevens, 1972; Murray and Arnott, 1993). The potential for the connection between these emotional aspects of language and those found in music has already been discussed extensively and convincingly elsewhere (e.g., Juslin and Laukka, 2003). As such, our focus here is on non-emotional aspects of language. However, it would be negligent on our part to avoid discussing emotional prosody entirely.

As an example of these connections, experience with music is tied to success in decoding prosodic aspects of language. There is a close correlation between prosody and emotional processing (Bänziger and Scherer, 2005; Grandjean et al., 2006), including an intriguing tendency for specific musical intervals (particularly the minor third) to emerge in emotional or stylized aspects of language (Curtis and Bharucha, 2010; Day-O’Connell, 2013). Prosody/emotion links suggest that advantages in prosodic processing may account for enhanced emotional processing abilities by musically trained adults (Thompson et al., 2004; Lima and Castro, 2011; Pinheiro et al., 2015; though cf. Trimmer and Cuddy, 2008). On the other end of the continuum of musical abilities, adults with congenital amusia exhibit a deficit in identifying emotional expressions in speech based on prosodic cues (Patel et al., 1998b, 2008; Jiang et al., 2012; Thompson et al., 2012).

More generally, it is often said to be the case that appreciation of music is derived in part from its emotional content (Juslin and Laukka, 2003; Juslin et al., 2008; Salimpoor et al., 2009). This emotional content has been argued to be universally accessible even across cultures (Fritz et al., 2009). Just as there are structural properties that appear to be shared across musical styles, there are also commonalities in how emotions are conveyed through music (Brown and Jordania, 2011). These bear many similarities to expressions of emotion in prosody (Juslin and Laukka, 2003). In one such parallel, infant-directed speech, also known as “motherese,” has been claimed to primarily derive its status from uninhibited vocal emotion on the part of people addressing themselves to infants (Trainor et al., 2000). Likewise, infants and young children are also often sung to using unique vocal performance styles, such as the lullaby. Perhaps musical content gains its emotional valence in part through the same mechanisms that allow for the decoding of speech prosody.

### Tying Up Loose Ends

It is certainly the case that the emotional links between music and prosody are important; however, as we have tried to spotlight, prosody is used for more in language than just emotion. Indeed, several aspects of prosody that are independent of emotion also gain greater salience under our present account. Although non-emotional aspects of prosodic perception have been less frequently studied than emotional prosody, Moreno et al. (2009) found that 8-year-olds with six months of musical training were more accurate than children without musical training at detecting small pitch excursions at the end of simple sentences; this was similar to previous results obtained for adults (Schön et al., 2004; Marques et al., 2007). This aligns with a version of the OPERA hypothesis (Patel, 2014), which is a framework that outlines how and why musical training might improve performance in other auditory domains. Under the OPERA hypothesis, speech processing is enhanced in musicians when speech and music both share certain cognitive resources, but music taxes those resources more than language does. Perhaps music is more demanding on prosodic structure-building than language. This in many ways resembles the perspective of Besson et al. (2011), who contrasted performance improvements on phonetic tasks that they claimed relied on shared auditory resources versus performance improvements that were the result of training transfer.

An additional example of a relevant non-emotion-related finding is the correlation between how variable the duration of a language’s segments are (as measured using nPVI—the normalized Pairwise Variability Index—or other indices of vowel and consonant duration, such as the coefficient of variation) and how variable the length of different notes in that culture’s music is. Patel and colleagues (Huron and Ollen, 2003; Patel and Daniele, 2003; Patel, 2005; Patel et al., 2006) found that, just as English speech has more variable segment durations than French speech, instrumental music from English composers has more variable note durations than music from French composers. Similar correlations exist among different dialects of English, as revealed from recordings made in the mid-twentieth century in the United States, Ireland, and Scotland (McGowan and Levitt, 2011). Under a prosodic account of musical structure, the reason for the correlation is quite clear: if musical structure and prosodic structure are closely linked, it makes sense for the statistical properties of the music within a culture to echo those of the prosodic structure.

Another prosodic process that might interact with musical experience is word segmentation, the process of locating word boundaries in fluent speech. Word segmentation is in part prosodic, as prosodic words are a part of the prosodic hierarchy. If prosody is linked to musical structure, it might then make sense for more musically experienced children to outperform their peers at tasks of word segmentation, as segmenting diverse types of musical streams might translate to the ability to segment auditory speech streams into prosodic words. And, indeed, this is exactly what was found for 8-year-old children on a test of their statistical learning proficiency (François et al., 2013).

Relatedly, modeling approaches that have made use of transitional probabilities have been successful in modeling perceived musical expectedness ratings. In one experiment testing this, notes that had higher transitional probabilities with regard to their context were rated as more expected and elicited different electrophysiological responses from notes that had lower transitional probabilities (Pearce et al., 2010). Transitional probabilities are, by their nature, non-recursive. Although evidence for statistical learning has occasionally been demonstrated across long distances (Hsu et al., 2014; Kabdebon et al., 2015), this long-distance learning typically requires strong inter-unit similarity between the units that form the long-distance dependencies (Gebhart et al., 2009). These constraints on which units can show long-distance statistical learning are reminiscent of the co-occurrence restrictions in systems such as vowel harmony (e.g., vowel harmony applies in a long-distance fashion across adjacent vowels, “skipping over” consonants). Although it may be possible to incorporate ideas of statistical learning under the purview of syntax, the findings take on new significance under a prosodic account, as statistical learning has been clearly shown to be relevant for prosodic processes such as word segmentation (Saffran et al., 1996).

A final group of results that are parsimoniously explained by a prosodic account of musical structure are auditory illusions. Consider the speech-to-song illusion (Deutsch et al., 2011). In the speech-to-song illusion, certain phrases (in Deutsch et al., 2011, “sometimes behave so strangely”) when repeated numerous times suddenly begin to sound less like speech and more like music. This transformation is accompanied by increases in the salience of the pitch contour to listeners and, after being asked to repeat the phrase in question, by increasing faithfulness of such repetitions to musical intervals. It appears likely that the repetition of the prosodic information of the sentence are what allows it to begin to take on the structural characteristics of a song.

Another frequently discussed auditory illusion is the tritone paradox, where tritone pairs (i.e., tone pairs separated by a half-octave) are variably perceived as increasing or decreasing in pitch depending on, for example, the dialect of the listeners (Deutsch, 1997). Intriguingly, which pairs are perceived as increasing and which are perceived as decreasing is correlated with the pitch range of the perceiver’s speech (Deutsch et al., 1990). Forging a direct link between a speaker’s prosodic abilities and musical abilities makes the link clearer: the prosody of one’s native dialect would help determine which pitch ranges and intervals a speaker was most used to, and would therefore affect their perception of those intervals outside of the domain of language.

## The Nature of a Music/Prosody Connection

Having established, then, that music and prosody are connected, this leads to the question: *how* are they connected? We propose that there are four possibilities, although not all of these possibilities seem equally likely. First, musical structure and prosodic structure might be functionally independent but parallel. That is, the similarities between them might either be coincidental or based on some underlying structural organization of the brain. Second, the use of musical structure and prosodic structure may involve similar underlying resources, but could differ in some aspects of their implementation. Third, certain aspects of musical structure might be “fundamental”, while prosodic structure depends on it. Finally, prosodic structure might be “fundamental”, while certain aspects of musical structure grew up around it.

First, let us address the idea that the structural properties of prosody and music are similar but related only coincidentally, not because of any particular shared mechanism. The first, “coincidental,” argument relies on the premise that prosody and music might have similar structural properties, but do not share underlying structural units. We believe that there are simply too many similarities between the processing of music and the processing of prosody for this fact to be simply an accident of mental organization. Why, under a “coincidental” account, should children or adults with musical training be better at detecting pitch excursions or segmenting words in speech (Schön et al., 2004; Moreno et al., 2009; François et al., 2013; Patel, 2013; Tillmann, 2014) or should adults with congenital amusia be worse at detecting emotional prosody in speech (Thompson et al., 2012)?

The second, “passive,” argument would be based on the idea that musical structure and prosodic structure do have similarities, but that those similarities are entirely due to a third factor. It behooves someone selecting this option, then, to find the third factor that explains the similarities between the modes of processing, such as cognitive control (see Slevc and Okada, 2015). Unfortunately, though, prosody is not a frequent object of study with regard to more domain-general cognitive functions, so it seems that this argument awaits further data.

The latter two other hypotheses represent more direct (and more radical) ways that prosody and music might be interrelated. It will likely be exceedingly difficult to resolve between these two hypotheses, given how difficult music and language are to analyze in the fossil and evolutionary anthropological record (Fitch, 2000; Hauser et al., 2002; Hauser and McDermott, 2003); however, this has not prevented many researchers from trying (Wallin et al., 2000). Nevertheless, if either prosody underlies music or music underlies prosody, then we can develop numerous predictions for the parallels between musical structure and prosodic structure, as the development and deployment of one ability marches in tandem with the other. Under the third argument outlined above, prosodic structure follows from musical structure. Musical structure forms the underlying basis of the organization that we observe within prosodic units. This bears some similarities to, say, evolutionary accounts of language that put music as the origin of linguistic abilities (Mithen, 2007). This may explain, for example, why talkers’ utterances trade off at consonant intervals during conversations with perfectly shared information but trade off at dissonant intervals during conversations with imperfectly shared information (Okada et al., 2012), or why the “stylized interjection” (i.e., the “calling contour”) is often associated with a minor third interval (Day-O’Connell, 2013).

The opposite notion is equally persuasive. It might be the case that the development of musical structure followed from prosodic structure. Linguistic prosody bears many similarities to the sorts of vocal signals that other animals produce emotionally (Juslin and Laukka, 2003). And prosodic structure seems to be closely linked to syntactic structure (Selkirk, 1984; Langus et al., 2012), which many theorists take as the crux of what a human language is (Hauser et al., 2002). Thus, while it is almost impossible to say for certain whether music or prosody preceded the other, in light of how closely related syntax and prosody are and accordingly how central and unique recursive (syntactic) thought is to human nature, we err on the side of the notion that prosody arrived on the scene first, providing a framework and fertile ground upon which music could grow and organize itself, perhaps (though not necessarily) capitalizing on the emotional or expressive aspects of prosodic structure (Panksepp, 2009). Under this account, then, music still continues, with some variation, to make use of that structure.

## Connections

### Roles for Both Prosody and Phonetics

Prosody, of course, is not entirely separable from other domains of language perception. Consider the perception of segmental phonetics, that is, speech when considered at the level of individual sounds. Musical pitches, like speech sounds, show evidence of categorical perception in trained listeners. Musicians (and, to a lesser extent, non-musicians) show sharp identification curves when asked to characterize musical intervals, with very good discriminability across category boundaries but only middling discriminability within single categories (Burns and Ward, 1978; Zatorre and Halpern, 1979). Categorical perception of musical intervals has been localized to a similar (although right-lateralized rather than left-lateralized) brain region as the categorical perception of speech sounds (Klein and Zatorre, 2011). This pattern was not found for a pitch discrimination task that did not entail categorical perception (Klein and Zatorre, 2015).

Trained musicians perform broadly better than non-musicians at phonetic perception tasks across a variety of measures. For example, musicians show stronger categorical perception for speech sounds than non-musicians do, with the strength of that categorical perception corresponding to higher-fidelity subcortical encoding of speech sounds (Bidelman et al., 2014). In one comprehensive test of second language abilities comparing groups of musicians to non-musicians, musically trained second language learners of English scored higher on measures of phonetic and phonological learning—but not any higher-level linguistic abilities, such as syntactic grammaticality judgments or vocabulary size—than non-musically trained learners (Slevc and Miyake, 2006). As such, it is plausible to suggest an analogy between music and sound-based linguistic systems in lieu of the syntactic explanation of musical structure. Yet while a parallel with phonetic processing may explain these findings and some of the interactions apparent in early music and language processing, a phonetic account is not sufficient to explain many of the findings of a higher-level relationships between music and language processing described earlier (e.g., Patel et al., 1998a; Koelsch, 2005; Koelsch et al., 2013).

An increased focus on the ties between musical structure and prosodic structure does not preclude the idea that musical processing aids the perception of individual segments as well. Perhaps one way in which these abilities tie in to the suprasegmental nature of prosody is related to the perception of lexical tone. Like prosody, lexical tone is suprasegmental, as it relies on pitch contours. However, like segmental information, it is realized on individual phonetic segments, such as vowels, and is related to the expression of basic lexical distinctions. Musicians who speak a non-tonal language are better than their non-musician peers at detecting tonal variation in languages that use lexical tone (Wong et al., 2007; Delogu et al., 2010), in a way reminiscent of tonal language speakers’ increased proficiency with musical pitch discrimination (Bidelman et al., 2013). Exploring segmental contrasts that are similar to prosody is one way in which the relationship between prosody, phonetics, and music may be related.

### Roles for Both Prosody and Syntax

Despite reasons to posit links between musical and prosodic structure, there is still evidence that music can interact with linguistic syntactic manipulations that are divorced from prosody, and that prosody may not overlap with music. For example, Rogalsky et al. (2011) found that comparing (auditory) scrambled sentences (presumably with disrupted prosody and syntax) to intact sentences reveals brain regions that largely do not correspond to those that are activated by music. Those regions that do respond to both music and prosody seem to be largely related to lower-level amplitude modulation, not to higher-level structural processes. This suggests non-overlap between prosodic brain regions and musical brain regions, at least in regions involved in this particular comparison of music to language.

Other evidence for musical relationships with syntax that are not attributable to prosody come from studies finding music/syntax interactions using isochronous word-by-word (and chord-by-chord) presentation (Koelsch et al., 2005; Steinbeis and Koelsch, 2008; Carrus et al., 2013), as this removes the timing, loudness, and pitch cues necessary to produce prosodic structure (though note that these prosodic cues do seem to be better exploited by musicians than non-musicians; Besson et al., 2011). These studies rely on the interpretation of particular ERP components that are traditionally associated with syntax, especially the LAN and P600. Although the syntactic natures of both the LAN (Steinhauer and Drury, 2012) and the P600 (Van Herten et al., 2005; Martín-Loeches et al., 2006) have been brought into question, this still provides support for a tie between music and syntax.

Therefore, we suggest that the increased focus on prosodic structure that we recommend in this paper does not necessitate an abandonment of syntactic structure as a potential analog for at least some components of music. Music is a complex system that may have multiple parallels in the similarly complex structures of language. And, given the many parallels between the structures of prosody and those of syntax, it is likely that finding ways to isolate the two will require careful study. However, we entreat researchers studying the ties between music and language to more carefully consider the possibility that it is in prosody, not simply in syntax, where parallels lie.

## Predictions

Encouraging renewed interest in prosodic structure as an analog to musical structure does more than just suggest a convenient way to tie together diverse studies that have examined both music and language. It also allows for a rich set of predictions that could be useful to further work in elucidating parallels between these domains.

For example, it should be possible to show further enhancements for musically trained participants in their perception of prosodic information, echoing similar advantages associated with phonetic processing. Examples of the latter include MEG evidence that French speaking musicians showed stronger mismatch negativity (MMN) responses to duration-related mismatches in non-linguistic stimuli than non-musician French speakers and even than non-musician Finnish speakers, who use contrastive consonant and vowel information in their own language (Marie et al., 2012). These advantages may be related to differences in the left planum temporale (PT) that are evident when comparing musicians to non-musicians (Elmer et al., 2012, 2013).

Given these advantages in phonetic processing, along with the prosody/music parallels discussed so far, we would also expect musicians to have improved prosodic processing abilities. There is indeed considerable evidence for such a relationship with emotional prosody (Thompson et al., 2004; Trimmer and Cuddy, 2008; Lima and Castro, 2011; Pinheiro et al., 2015). However, prosodic perception is more than just emotional processing. A prosody/music link predicts that musicians should also show advantages in prosodic abilities unrelated to emotional contexts. There is already some evidence for non-emotional prosodic enhancements in musicians; for example, musically trained children find it easier than non-musically trained children to extract statistical regularities they can use to determine word boundaries (François et al., 2013). Musically trained participants have more veridical neurophysiological representations of speech sounds for both children (Chobert et al., 2014) and adults (Wong et al., 2007). Musicians are also more sensitive to subtle fundamental frequency (F0) changes in speech when compared to non-musicians (Schön et al., 2004) which could be the result of more robust fundamental frequency tracking in their auditory brainstem responses (Musacchia et al., 2007).

This account predicts that musicians’ advantages should also extend to more subtle aspects of prosodic processing. Dilley and Heffner (2013) moved F0 peaks through utterances in 30 ms increments in a way that they hoped would evoke prosodic boundaries. They had participants perform a variety of tasks, including discriminating between two versions of the same utterance, repeating different versions of each utterance, and explicitly judging word stress, all meant to assess categorical perception of the location of prosodic prominences within the sentences. In general, they found that listeners perceived the differences between the locations of possible prominence categorically. That is, just like what has been abundantly demonstrated in the perception of individual speech sounds (Liberman et al., 1957), listeners’ judgments of prosodic contrasts showed strong evidence for the influence of prosodic categories on the basic perception of those contrasts. Put another way, participants judged changes in the linear continuum non-linearly. However, there were strong individual differences in how categorical (i.e., how non-linear) participants’ judgments actually were. Perhaps participants’ musical training is one factor to explain the variation between individuals. Given the importance of prosody to determinations of foreign accent (Munro and Derwing, 2001; Trofimovich and Baker, 2006), musicians may be better able to replicate the prosodic properties of a second language that they are trying to learn, and might therefore sound less accented to an L1 speaker (Slevc and Miyake, 2006).

This hypothesis could additionally relate to the development of musical and linguistic skills. Given the conjecture that prosodic structure and musical structure have shared underpinnings, prosodic abilities should correlate with musical abilities across development. Indeed, that is what is generally found; both abilities develop quite early in life and show similar developmental trajectories (Brandt et al., 2012). Infants develop a very early preference, at as young as one month of age, for infant-directed speech, with accompanying pitch, volume, and duration cues (Fernald and Kuhl, 1987; Cooper and Aslin, 1990). Similarly, infants also prefer infant-directed singing over adult-directed singing (Trainor, 1996). Children as young as six to nine months prefer the prosody of their native language (Jusczyk et al., 1993; Höhle et al., 2009), and can productively use that prosodic information to help them segment words and phrases (Soderstrom et al., 2003). At around the same time period that they are learning the prosodic structures of their nature language, they also begin to show evidence for a preference for native musical structures. Infants of a similar age show sensitivity to the musical structures used around them (Krumhansl and Jusczyk, 1990; Jusczyk and Krumhansl, 1993), prefer the musical structures of familiar musical styles to those of unfamiliar ones (Soley and Hannon, 2010), and can recognize brief, familiar musical passages (Saffran et al., 2000).

Clinical populations might also help test these hypotheses. Dysprosody, the clinical loss of prosodic characteristics of speech, is an infrequent condition that is typically acquired at some point during adulthood (Whitty, 1964; Samuel et al., 1998; Sidtis and Van Lancker Sidtis, 2003). However, dysprosody in language production is often associated with neurodegenerative diseases such as Parkinson’s disease, suggesting the production or perception of prosody may be associated with the basal ganglia and dopaminergic pathways within the brain (Caekebeke et al., 1991; Van Lancker Sidtis et al., 2006; Skodda et al., 2009). Although the roots of dysprosody in this population may reside with difficulties in production, perceptual prosodic capacities among individuals with Parkinson’s disease are currently understudied. One investigation found that deficits in the perception of the emotional content of music did not seem to be strongly correlated with deficits in the perception of emotional speech prosody in Parkinson’s patients (Lima et al., 2013); nevertheless this framework predicts that patients with perceptual dysprosody should also suffer from deficits in perception and production of musical structure.

Similarly, people with schizophrenia are often said to have problems with the comprehension of prosody. This is particularly true for the comprehension of affective prosody, which is very well-studied in people with schizophrenia (Murphy and Cutting, 1990; Mitchell et al., 2004; Hoekert et al., 2007; Roux et al., 2010), and is connected to concurrent emotional face processing deficits (Edwards et al., 2001, 2002). Fewer studies have investigated prosodic processing in schizophrenia beyond emotion; those that have, though, have also found deficits in more general prosodic processing (Leitman et al., 2005; Matsumoto et al., 2006). Given these results, it may also be the case that people with schizophrenia show impaired perception of musical structure. So too with people with depression, who display differences from typical controls in the production (Garcia-Toro et al., 2000) and perception (Uekermann et al., 2008) of prosody. Intriguingly, they also show differences in activation within brain regions typically associated with reward from control participants when listening to their favorite songs (Osuch et al., 2009).

## Conclusion

Much of the literature on the structural parallels between language and music has focused on syntax. Although this is understandable in light of the focus within the language sciences on syntactic structure, it leaves relatively understudied another important aspect of language: the prosodic patterns of loudness, pitch, and timing that make up the rhythms and melodies of speech. It is possible that, when creating the *Hallelujah* chorus, Handel wept for joy solely because of the pleasant syntactic structure of the music he was creating. But, if it is the case that music and language are entwined closely enough to share crucial structural ingredients, it seems more likely (at least to us) that the pleasure of music might be enabled by the acoustic characteristics shared between music and prosody. It might be the case that the speech-to-song illusion (Deutsch et al., 2011) occurs because of the repetition of the syntactic structure in the simple utterances that characterize the illusion; however, it at least appears to be more direct to see it as such because of the repeated prosodic patterns of pitch, timing, and volume present in every utterance.

Prosody has a structure of its own, independent from (but interwoven with) the syntactic structure of language. Recalling the initial speculation found in Lerdahl and Jackendoff (1983), we argue here that the relationship of musical structure to language may gain much from discussion in terms of prosodic structure. This helps tie together several previous strands of research related to language/music parallels, and allows for several novel predictions about the relationship between prosodic processing in language and music processing. Syntax is, without a doubt, a critical part of language processing. And to consider prosody as a potential analog of musical structure does not require abandoning the syntactic parallels that have been described. But ignoring the rest of language when seeking the most analogous processes to music within language is an approach that, we believe, has proven misguided.

## Conflict of Interest Statement

The authors declare that the research was conducted in the absence of any commercial or financial relationships that could be construed as a potential conflict of interest.

## Footnotes

1 “Ambisyllabic” consonants are a proposed exception to this rule (Schiller et al., 1997), and under some theories a lower-level item (such as a single phoneme) might not be assigned to an individual syllable. However, their psychological status remains unclear. It is worth noting that notes that seem to straddle two phrases are also found sporadically in music (Lerdahl and Jackendoff, 1983).
